# Theoretical study of the kinetics of chlorine atom abstraction from chloromethanes by atomic chlorine

**DOI:** 10.1007/s00894-013-1779-y

**Published:** 2013-03-02

**Authors:** Katarzyna Brudnik, Maria Twarda, Dariusz Sarzyński, Jerzy T. Jodkowski

**Affiliations:** Department of Physical Chemistry, Wroclaw Medical University, pl. Nankiera 1, 50-140 Wroclaw, Poland

**Keywords:** Chemical kinetics, Gas-phase reactions, Reaction mechanism, Chlorine abstraction, Chloromethanes

## Abstract

Ab initio calculations at the G3 level were used in a theoretical description of the kinetics and mechanism of the chlorine abstraction reactions from mono-, di-, tri- and tetra-chloromethane by chlorine atoms. The calculated profiles of the potential energy surface of the reaction systems show that the mechanism of the studied reactions is complex and the Cl-abstraction proceeds via the formation of intermediate complexes. The multi-step reaction mechanism consists of two elementary steps in the case of CCl_4_ + Cl, and three for the other reactions. Rate constants were calculated using the theoretical method based on the RRKM theory and the simplified version of the statistical adiabatic channel model. The temperature dependencies of the calculated rate constants can be expressed, in temperature range of 200–3,000 K as

$$ \begin{array}{*{20}c} {k\left( {\mathrm{C}{{\mathrm{H}}_3}\mathrm{C}\mathrm{l}+\mathrm{Cl}} \right) = 2.08\times {10^{-11 }}\times {{{\left( {{{\mathrm{T}} \left/ {300 } \right.}} \right)}}^{1.63 }}\times\exp \left( {{-12780 \left/ {\mathrm{T}} \right.}} \right)\ }{\mathrm{c}{{\mathrm{m}}^3}\mathrm{molecul}{{\mathrm{e}}^{-1 }}{{\mathrm{s}}^{-1 }}} \\ {k\left( {\mathrm{C}{{\mathrm{H}}_2}\mathrm{C}{{\mathrm{l}}_2}+\mathrm{Cl}} \right) = 2.36\times {10^{-11 }}\times {{{\left( {{{\mathrm{T}} \left/ {300 } \right.}} \right)}}^{1.23 }}\times\exp \left( {{-10960 \left/ {\mathrm{T}} \right.}} \right)}{\mathrm{c}{{\mathrm{m}}^3}\mathrm{molecul}{{\mathrm{e}}^{-1 }}{{\mathrm{s}}^{-1 }}} \\ {k\left( {\mathrm{C}\mathrm{HC}{{\mathrm{l}}_3}+\mathrm{Cl}} \right) = 5.28\times {10^{-11 }}\times {{{\left( {{{\mathrm{T}} \left/ {300 } \right.}} \right)}}^{0.97 }}\times\exp \left( {{-9200 \left/ {\mathrm{T}} \right.}} \right)}{\mathrm{c}{{\mathrm{m}}^3}\mathrm{molecul}{{\mathrm{e}}^{-1 }}{{\mathrm{s}}^{-1 }}} \\ {k\left( {\mathrm{C}\mathrm{C}{{\mathrm{l}}_4}+\mathrm{Cl}} \right) = 1.51\times {10^{-10 }}\times {{{\left( {{{\mathrm{T}} \left/ {300 } \right.}} \right)}}^{0.58 }}\times \exp \left( {{-7790 \left/ {\mathrm{T}} \right.}} \right)}{\mathrm{c}{{\mathrm{m}}^3}\mathrm{molecul}{{\mathrm{e}}^{-1 }}{{\mathrm{s}}^{-1 }}} \\ \end{array} $$

The rate constants for the reverse reactions CH_3_/CH_2_Cl/CHCl_2_/CCl_3_ + Cl_2_ were calculated via the equilibrium constants derived theoretically. The kinetic equations

$$ \begin{array}{*{20}c} {k\left( {\mathrm{C}{{\mathrm{H}}_3}+\mathrm{C}{{\mathrm{l}}_2}} \right) = 6.70\times {10^{-13 }}\times {{{\left( {{{\mathrm{T}} \left/ {300 } \right.}} \right)}}^{1.51 }}\times \exp \left( {{270 \left/ {\mathrm{T}} \right.}} \right)}{\mathrm{c}{{\mathrm{m}}^3}\mathrm{molecul}{{\mathrm{e}}^{-1 }}{{\mathrm{s}}^{-1 }}} \\ {k\left( {\mathrm{C}{{\mathrm{H}}_2}\mathrm{C}\mathrm{l}+\mathrm{C}{{\mathrm{l}}_2}} \right) = 7.34\times {10^{-14 }}\times {{{\left( {{{\mathrm{T}} \left/ {300 } \right.}} \right)}}^{1.43 }}\times \exp \left( {{390 \left/ {\mathrm{T}} \right.}} \right)}{\mathrm{c}{{\mathrm{m}}^3}\mathrm{molecul}{{\mathrm{e}}^{-1 }}{{\mathrm{s}}^{-1 }}} \\ {k\left( {\mathrm{C}\mathrm{HC}{{\mathrm{l}}_2}+\mathrm{C}{{\mathrm{l}}_2}} \right) = 6.81\times {10^{-14 }}\times {{{\left( {{{\mathrm{T}} \left/ {300 } \right.}} \right)}}^{1.60 }}\times \exp \left( {{-370 \left/ {\mathrm{T}} \right.}} \right)}{\mathrm{c}{{\mathrm{m}}^3}\mathrm{molecul}{{\mathrm{e}}^{-1 }}{{\mathrm{s}}^{-1 }}} \\ {k\left( {\mathrm{C}\mathrm{C}{{\mathrm{l}}_3}+\mathrm{C}{{\mathrm{l}}_2}} \right) = 1.43\times {10^{-13 }}\times {{{\left( {{{\mathrm{T}} \left/ {300 } \right.}} \right)}}^{1.52 }}\times \exp \left( {{-550 \left/ {\mathrm{T}} \right.}} \right)}{\mathrm{c}{{\mathrm{m}}^3}\mathrm{molecul}{{\mathrm{e}}^{-1 }}{{\mathrm{s}}^{-1 }}} \\ \end{array} $$

allow a very good description of the reaction kinetics. The derived expressions are a substantial supplement to the kinetic data necessary to describe and model the complex gas-phase reactions of importance in combustion and atmospheric chemistry.

## Introduction

Chlorinated alkanes are used widely in laboratory syntheses and in the chemical industry [1]. As a consequence, they are penetrating into the environment in steadily increasing amounts. Chloroalkanes and products of their environmental reactions are considered toxic and biocumulative species. The chemical inertness and high volatility of chloromethanes mean that they can remain in the atmosphere for a very long time. The products of the atmospheric destruction of chloromethanes have been proven to have a significant impact on chlorine chemistry in the atmosphere and may be involved in various catalytic reaction cycles responsible for the depletion of the stratospheric ozone layer [1, 2].

Monochloromethane (CH_3_Cl) is regarded as the most abundant halocarbon in the troposphere, and the largest natural source of stratospheric chlorine [1, 3]. The variability of CH_3_Cl in the ice core over the last two millennia suggests a relationship between the concentration of atmospheric CH_3_Cl and global mean temperature, which indicates the possibility that a warmer future climate may result in higher tropospheric CH_3_Cl levels. Major sources of CH_3_Cl are biomass burning, oceanic emissions and vegetative emissions. The other chloromethanes occurring in polluted atmosphere, i.e., dichloromethane (CH_2_Cl_2_), trichloromethane (CHCl_3_) and tetrachloromethane (CCl_4_), are released primarily from industrial processes [1–5].

The reaction with hydroxyl radical is a major loss pathway for atmospheric chloromethanes. The reactions of Cl atoms with many haloalkanes can also become of some importance because the rate constants for these reactions are distinctly higher than those for the corresponding reactions with OH radicals. Therefore, in the marine boundary layer and in polar regions where the concentration of chlorine atoms is significant, Cl-initiated reactions may play a key role in the decay of many organic compounds in the troposphere.

The sources of chlorine atoms are the photochemically labile chlorine compounds such as Cl_2_ and ClNO_2_ produced in some aqueous-phase reactions in airborne seawater droplets [1, 2]. Chlorine atoms and their oxides are highly reactive species and can profoundly affect atmospheric composition. The gas-phase reactions of chlorine atoms with the hydrogen-containing atmospheric halocarbons lead to the facile generation of the corresponding free radicals via hydrogen atom abstraction. These reactions also play an important role in the processes of industrial chlorination and incineration of hazardous halogenated wastes, and their kinetics have been the subject of many theoretical and experimental studies [6–8]. Considerably less recognized are the kinetics of chlorine abstraction reactions. To the best of our knowledge, these reactions have not been investigated experimentally. In this study, we present a theoretical analysis of the mechanism and kinetics of the reactions of chloromethanes, CH_4−x_Cl_x_ (x = 1,2,3 and 4) with atomic chlorine:

1 $$ \mathrm{C}{{\mathrm{H}}_{4-x }}\mathrm{C}{{\mathrm{l}}_x}+\mathrm{C}\mathrm{l}\to \mathrm{C}{{\mathrm{H}}_{4-x }}\mathrm{C}{{\mathrm{l}}_{x-1 }}+\mathrm{C}{{\mathrm{l}}_2} $$

Kinetic information on the reactions indicated in Eq. (1) is very limited. Chlorine abstraction reactions proceed incomparably slower than the analogous H-abstraction processes, and grow in importance only at high temperatures. The kinetics of the reverse processes

-1 $$ \mathrm{C}{{\mathrm{H}}_{{4-\mathrm{x}}}}\mathrm{C}{{\mathrm{l}}_{{\mathrm{x}-1}}}+\mathrm{C}{{\mathrm{l}}_2}\to \mathrm{C}{{\mathrm{H}}_{{4-\mathrm{x}}}}\mathrm{C}{{\mathrm{l}}_{\mathrm{x}}}+\mathrm{C}\mathrm{l} $$

between chloromethyl radicals and molecular chlorine is considerably better known [6–8]. The reactions indicated in Eq. (−1) indeed also represent chlorine abstraction processes; however, Cl atoms in a molecule of Cl_2_ are bound distinctly weaker in comparison with those in chloromethane. In consequence, the reverse reactions (Eq. −1) are relatively fast processes at ambient temperature and their kinetics have been studied experimentally over a wide temperature range, allowing comparison of experimental results with the theoretical kinetic results obtained in this study.

## Computational details

It is well known that the G2 method [9] well reproduces the structural parameters and molecular properties of a wide group of organic compounds. The haloalkanes were studied theoretically using quantum mechanical ab initio methods at various levels of theory. Reliable values relating to thermochemical properties and vibrational frequencies have been obtained using G2 methodology for perhalogenated methanols, methyl hypohalites, halogenated alkyl and alkoxy radicals [10–20]. The G2 method was also used successfully in the theoretical description of the kinetics and mechanism of the abstraction of hydrogen from methanol by halogen atoms [21–23]. We decided to use the G3 method [24] in our investigations. This method provides the same quality of computational results but with a considerable reduction in the time and the levels of calculations required in comparison with the G2 method. All quantum mechanical ab initio calculations were carried out using the Gaussian 09 program package [25]. The geometries of all stationary point structures of the potential energy surface were fully optimized at both the SCF and MP2 levels with the 6–31G(d) basis set. Relative total energies were examined using G3 methodology [24].

The rate constants of the reactions studied were analyzed in terms of conventional transition state theory (TST) [26, 27] according to the equation

2 $$ {{\mathrm{k}}_{\mathrm{T}\mathrm{ST}}}={\upkappa_{\mathrm{T}}}\sigma \frac{{{{\mathrm{k}}_{\mathrm{B}}}\mathrm{T}}}{\mathrm{h}}\exp \left( {\frac{{\varDelta {{\mathrm{S}}^{\ne }}}}{\mathrm{R}}} \right)\exp \left( {-\frac{{\varDelta {{\mathrm{H}}^{\ne }}}}{\mathrm{R}\mathrm{T}}} \right) $$

where κ_T_ is the tunneling correction factor, *σ* denotes a symmetry factor related to degeneracy of the reaction path, and k_B_ and *h* are the Boltzmann and Planck constants, respectively. Δ*S* ^≠^ is the activation entropy and Δ*H* ^≠^ the activation enthalpy of the reaction under investigation. Values of κ_T_ were evaluated from the simple Wigner’s expression [26]

3 $$ {\upkappa_{\mathrm{T}}}\cong 1-\frac{1}{24 }{{\left( {\frac{{\mathrm{h}{\upnu^{\ne }}}}{{{{\mathrm{k}}_{\mathrm{B}}}\mathrm{T}}}} \right)}^2} $$

with the imaginary frequencies *ν* ^≠^ of the transition state obtained in the geometry optimization performed at a higher level of theory, i.e., from MP2/6–31G(d) calculations. The vibrational and rotational contributions to the thermodynamic functions were derived by classical harmonic-oscillator rigid-rotor approximation (no free or internal rotation was considered).

## Results and discussion

**Fig. 1:**
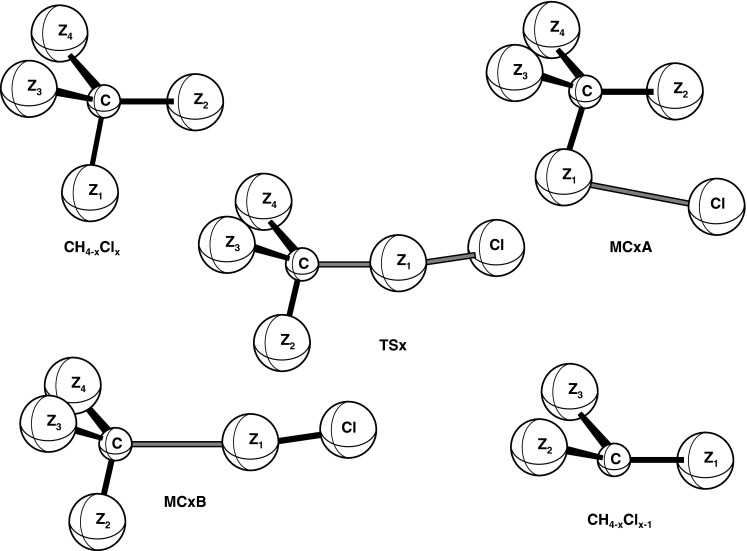
Definition of the geometrical parameters of molecular structures taking part in the mechanism of reaction of atomic chlorine with CH_3_Cl, CH_2_Cl_2_, CHCl_3_ and CCl_4_; *Z* denotes H or Cl atoms, and *x* = 1, 2, 3 or 4

The theoretical investigation of hydrogen abstraction from halomethanes by chlorine atoms shows that the mechanism of these reactions appears to be complex and consists of some consecutive elementary processes with the formation of loosely bound intermediate complexes [21–23]. Therefore, at each level of theory, the potential energy surface of the studied reactions was explored for the possible existence of transition states and intermediate complexes. The geometries of all structures were optimized fully and independently using analytical gradients at the SCF and MP2 levels with the 6–31G(d) basis set. The molecular arrangements and definitions of the structural parameters of the molecular structures taking part in the mechanism of reactions CH_4−x_Cl_x_ + Cl (x = 1,2,3 and 4) are shown in Fig. 1. The results of calculations including the geometrical parameters optimized at the MP2/6–31G(d) level, the harmonic vibrational frequencies, the rotational constants and the total G3(0 K) energies (ZPE included) for the reactants, products, intermediate complexes and transition states are given in Tables 1 and 2.

**Table 1:** Molecular properties of the reactants and products of the reactions under investigation calculated at the G3 level^a^. The vibrational frequencies *ν*_i_ (cm^−1^) obtained at the SCF/6–31G(d) level and scaled by 0.8929 (first column), derived in the MP2(full)/6–31G(d) calculations (second column) were scaled by 0.94 for reactants and products. Experimental frequencies [28–32] are given in parenthesis

	CH_3_Cl (C_3v_)			CH_2_Cl_2_ (C_2v_)			CHCl_3_ (C_3v_)			CCl_4_ (T_D_)		
C–Cl	1.7770			1.7673			1.7648			1.7688		
C–H	1.0877			1.0869			1.0859					
H–C–Cl	108.9063			108.2652			107.6497					
Cl–C–Cl				113.0095			111.2297			109.4712		
H–C–H	110.0290			110.7900								
A	157.84728			32.625180			3.284360			1.732250		
B	13.37535			3.291950			3.284350			1.732250		
C	13.37530			3.048430			1.703430			1.732250		
ν_1_	700	739	(732)	278	283	(282)	258	261	(261)	218	220	(217)
ν_2_	1,016	1,020	(1,017)	691	709	(717)	258	261	(261)	218	220	(217)
ν_3_	1,016	1,020	(1,017)	752	768	(758)	360	364	(363)	311	315	(314)
ν_4_	1,374	1,380	(1,355)	889	894	(898)	652	664	(680)	311	315	(314)
ν_5_	1,454	1,453	(1,452)	1,174	1,172	(1,153)	780	774	(774)	311	315	(314)
ν_6_	1,454	1,453	(1,452)	1,295	1,295	(1,268)	780	774	(774)	449	452	(459)
ν_7_	2,917	2,965	(2,937)	1,445	1,438	(1,467)	1,249	1,238	(1,220)	806	783	(776)
ν_8_	3,009	3,071	(3,039)	2,980	3,002	(2,999)	1,249	1,238	(1,220)	806	783	(776)
ν_9_	3,009	3,071	(3,039)	3,053	3,079	(3,040)	3,043	3,047	(3,034)	806	783	(776)
E_0_(G3)	−499.91251			−959.37103			−1,418.82886			−1,878.28323		

	CH_3_ (D_3h_)			CH_2_Cl (C_s_)			CHCl_2_ (C_s_)			CCl_3_ (C_3v_)		
C-Cl				1.7007			1.7029			1.7109		
C-H	1.0783			1.0780			1.0809					
H-C-Cl				117.4525			116.591					
Cl-C-Cl							119.0484			116.887		
H-C-H	120.0000			122.8962								
A	287.529180			275.216580			47.036420			3.377060		
B	287.529180			15.849680			3.350770			3.377030		
C	143.764590			15.012400			3.135160			1.699920		
ν_1_	275	380		441	313	(389)	290	302	(190 ± 50)	264	273	(290)
ν_2_	1,375	1,392	(1,428)	777	831	(829)	542	526		264	273	(290)
ν_3_	1,375	1,392	(1,428)	976	995		721	753	(845)	313	345	
ν_4_	2,933	3,028	(2,931)	1,381	1,407	(1,391)	863	891	(896)	484	494	(509)
ν_5_	3,090	3,206	(3,087)	2,996	3,074	(3,055)	1,228	1,247	(1,226)	894	896	(898)
ν_6_	3,090	3,206	(3,087)	3,127	3,215		3,063	3,113		894	896	(898)
E_0_(G3)	−39.79223			−499.25600			−958.71926			−1,418.18095		

^a^G3 molecular parameters: geometrical structure optimized at the MP2(Full)/6–31 G(d) level, (bond lengths in Å, valence and dihedral angles in degrees), rotational constants ABC in GHz, the total G3-energies in a.u. at 0 K (ZPE included)

**Table 2:** Molecular properties of structures taking part in the mechanism of reaction under investigation derived at the G3 level ^a^

	MC1A (C_s_)	TS1 (C_3v_)	MC1B (C_3v_)	MC2A	TS2 (C_s_)	MC2B (C_s_)	MC3A (C_s_)	TS3 (C_s_)	MC3B (C_s_)	TS4 (C_3v_)	MC4B (C_3v_)
ClCl_0_	3.1706	2.0952	2.0232	5.1473	2.1118	2.0232	3.4875	2.1344	2.0222	2.1589	2.0204
CCl_0_	1.7794	2.3218	3.0624	1.7675	2.2707	3.0366	1.7682	2.2175	3.0234	2.1747	3.0465
CH_1_	1.0880	1.0810	1.0791								
CCl_1_				1.7675	1.6944	1.6989	1.7634	1.7036	1.7014	1.7198	1.7104
CH_2_	1.0879	1.0810	1.0791	1.0866	1.0822	1.0793					
CCl_2_							1.7634	1.7036	1.7014	1.7198	1.7104
CH_3_	1.0879	1.0810	1.0791	1.0866	1.0822	1.0793	1.0857	1.0841	1.0818		
CCl_3_										1.7198	1.7104
ClH_3_	3.6039			3.3509			2.9519				
ClCl_0_C	92.418	180.000	180.000	56.503	176.405	177.145	83.245	177.0693	176.702	180.000	180.000
H_1_CCl_0_	108.622	98.310	93.025								
Cl_1_CCl_0_				112.976	109.557	107.229	111.111	106.6787	104.644	104.013	100.491
H_2_CCl_0_	108.761	98.310	93.025	108.277	96.591	91.559					
Cl_2_CCl_0_							111.111	106.6787	104.644	104.013	100.491
H_3_CCl_0_	108.761	98.310	93.025	108.277	96.591	91.559	107.289	94.7909	89.021		
Cl_3_CCl_0_										104.013	100.491
ClH_3_C				169.799			128.005				
H_1_CCl_0_Cl	180.000										
Cl_1_CCl_0_Cl				113.208	180.000	180.0	117.718	117.0209	117.052		
H_2_CCl_0_Cl	60.339			−126.932	119.938	61.020					
Cl_2_CCl_0_Cl							−117.718	−117.0209	−117.052		
H_3_CCl_0_Cl	−60.339			−6.705	−119.938	−61.020	0.000	0.000	0.000		
Cl_3_CCl_0_Cl											
A	14.037520	146.090730	143.959080	3.127490	18.545620	17.885810	2.114150	3.241100	3.192540	1.730210	1.703120
B	2.283420	2.280780	1.806480	0.851610	0.957270	0.744410	0.834620	0.807110	0.607000	0.627540	0.484290
C	1.988730	2.280780	1.806480	0.687030	0.916060	0.718320	0.762120	0.659880	0.518380	0.627540	0.484290
ν_1_	29	471i	53	5	501i	25	7	552i	24	612i	26
ν_2_	46	72	53	8	59	49	26	44	32	8	26
ν_3_	67	72	81	10	73	56	47	56	53	8	46
ν_4_	733	360	150	283	146	89	261	148	53	150	46
ν_5_	1,023	515	150	709	360	160	261	168	108	150	47
ν_6_	1,023	515	513	768	551	509	365	320	304	190	274
ν_7_	1,379	944	524	893	818	526	664	386	506	286	274
ν_8_	1,451	1,396	1,392	1,171	955	834	769	712	584	286	360
ν_9_	1,451	1,396	1,392	1,293	1,030	1,001	776	872	758	414	505
ν_10_	2,964	3,011	3,023	1,437	1,411	1,407	1,239	928	890	545	505
ν_11_	3,072	3,184	3,200	3,002	3,043	3,063	1,242	1,249	1,247	858	894
ν_12_	3,072	3,184	3,200	3,079	3,171	3,201	3,053	3,083	3,105	858	894
E_0_(G3)^b^	−15.15	105.88	100.66	−3.31	89.86	84.85	−6.72	75.07	66.99	62.15	44.90

^a^G3 molecular parameters: geometrical structure optimized at the MP2(Full)/6–31G(d) level, (bond lengths in Å, valence and dihedral angles in degrees), rotational constants ABC in GHz, the vibrational frequencies ν_i_ (cm^−1^) obtained at the MP2/6–31G(d) level and scaled by 0.940

^b^The total G3-energies in kJmol^−1^ at 0 K (ZPE included) calculated towards the G3-energy of the respective reactants’ energy

### Optimized molecular structures

The reactants are highly symmetrical molecular structures. A symmetry of point groups of C_3v_, C_2v_, C_3v_ and T_D_ is found for CH_3_Cl, CH_2_Cl_2_, CHCl_3_ and CCl_4_, respectively. The C–H and C–Cl bond lengths obtained in the geometry optimization performed at the MP2(Full)/6–31G(d) level for reactants are very close one to another. The lengths of C–Cl bonds in chloromethanes are within 1.765–1.777 Å whereas C–H bonds cover a narrow range of 1.086–1.088 Å. The differences in the values of the angular parameters, H–C–H and Cl–C–H in the reactants do not exceed 1°.

The radical products of the reactions under investigation show more visible differences in structural parameters. Methyl radical (CH_3_) is a planar structure with the symmetry of a D_3h_ point group. Trichloromethyl radical (CCl_3_) is also a highly symmetrical structure with a three-fold axis. The equilibrium geometry of CCl_3_ radical obtained at the MP2/6–31G(d) level corresponds to a C_3v_ symmetry. The other radicals, chloromethyl (CH_2_Cl) and dichloromethyl (CHCl_2_) are less symmetrical molecular structures. Either the C–H or the C–Cl bonds in the radical products are a little shorter than their counterparts in the parent chloromethanes. In contrast, the values of the angular parameters in radical products, Cl–C–H and H–C–H are distinctly greater than those in the corresponding reactants.

Except for tetrachloromethane, the attack of a chlorine atom on molecules of CH_3_Cl, CH_2_Cl_2_ and CHCl_3_ leads to the formation of the pre-reaction adducts, CH_4-x_Cl_x_…Cl (x = 1,2 and 3) denoted by MC1A, MC2A and MC3A for CH_3_Cl…Cl, CH_2_Cl_2_…Cl and CHCl_3_…Cl, respectively. The adducts MC1A and MC3A have a C_s_ symmetry, because the attacking chlorine atom is moving across the symmetry plane of the CH_3_Cl or CHCl_3_. The geometrical parameters of these molecular complexes retain the values that appear in the isolated reactants. All pre-reaction adducts, CH_3_Cl…Cl, CH_2_Cl_2_…Cl and CHCl_3_…Cl, are loose molecular structures with long contact distances between the attacking chlorine and reactant. The vibrational frequencies of MC1A, MC2A and MC3A from ν_4_ up are almost identical with the corresponding frequencies in the isolated reactants.

The transition states (CH_4-x_Cl_x-1_…Cl…Cl)^≠^, denoted by TSx (x = 1,2,3 and 4) describe the chlorine abstraction from CH_4−x_Cl_x_ by Cl atom. All transition states are reactant-like structures. In reactions CH_3_Cl + Cl and CCl_4_ + Cl, the attacking Cl atom is approaching CH_3_Cl and CCl_4_ along the C–Cl_0_ bond. The angle of C–Cl_0_–Cl is then equal to 180° and the three-fold axis of the reactant molecule (CH_3_Cl or CCl_4_) is retained in the structure of transition states TS1 and TS4, which have the symmetry of a C_3v_ point group. Both TS2 and TS3 are molecular structures with the C–Cl_0_/Cl_0_–Cl bonds breaking/formed located in the symmetry plane of the transition state. The attack of the chlorine atom at the TS2 and TS3 structures is nearly collinear, with values of the angle C–Cl_0_–Cl of 177° and 176° found for TS2 and TS3, respectively. The calculated lengths of the breaking bonds C–Cl_0_ are 30 % longer than those in the parent reactants. The length of C–Cl_0_ decreases with the increase in the number of chlorine atoms in the reactant molecule. The Cl_0_–Cl bonds formed are only 4–7 % longer than the Cl–Cl bond in molecular chlorine. The values of the other structural parameters of the transition states TSx are close to their counterparts in the isolated reactants.

The post-reaction adducts, CH_4−x_Cl_x−1_…Cl_2_, designated by MCxB (x = 1,2,3 and 4), are intermediates consisting of two subunits: radical CH_4−x_Cl_x−1_ and molecular chlorine Cl_2_, bonded in a molecular complex. All molecular complexes, MCxB keep the symmetry of their respective transition states TSx, so that MC1B and MC4B have a C_3v_ symmetry whereas MC2B and MC3B possess a C_s_ symmetry. The vibrational frequencies of these adducts are very close to the corresponding frequencies in the isolated radical product and Cl_2_.

In standard G3 approach, the vibrational frequencies are obtained in geometry optimization performed at the SCF/6–31G(d) level and scaled by 0.8929 to take into account their overestimation [24]. The vibrational frequencies derived in this way reproduce the experimental frequencies well and give a correct estimation of the zero-point energy of the reactants and products. However, the optimized structures for either intermediate complexes or transition states derived at the SCF and MP2 levels shows substantial differences in both geometrical parameters and vibrational frequencies. Therefore, our kinetic analysis of the studied reactions was based on the MP2(Full)/6–31G(d)-frequencies as the more credible ones. The value of the scaling factor for the MP2-frequencies was found by comparing the available experimental [28–33] and calculated MP2 frequencies for reactants and products. As can be seen from Table 1, using a value of the scaling factor of 0.940 leads to the best agreement of the MP2 and experimental frequencies [28–33]. Therefore, this scaling factor was used to calculate the vibrational frequencies of all molecular structures taking part in the mechanism of the studied reactions.

### Reaction energetics

The G3 method allows reliable estimation of the reaction energetics. The accuracy of these estimations based on the G3-energies is usually considered to be better than 6 kJ mol^−1^. The enthalpy of formation, $\varDelta H_{f,298}^0$ can be evaluated directly as the G3 enthalpy of the formation reaction of CH_x_Cl_y_ from the elemental reference compounds such as C_(g)_, H_2(g)_ and Cl_2(g)_, i.e., C_(g)_ + (x/2)H_2(g)_ + (y/2)Cl_2(g)_ → CH_x_Cl_y_, and taking into account that the elemental standard state of carbon is graphite. An alternative approach involved the total G3-energy for the atomization reaction CH_x_Cl_y_ → C_(g)_ + xH_(g)_ + yCl_(g)_ in combination with the calculated and experimental values of the enthalpy of formation of the gaseous atoms, such as $\varDelta H_{f,298}^0\left( {{{\mathrm{C}}_{{(\mathrm{g})}}}} \right)=716.7\;\mathrm{kJ}\;\mathrm{mo}{{\mathrm{l}}^{{\text{--} 1}}} \varDelta H_{f,298}^0$, $\varDelta H_{f,298}^0\left( {{{\mathrm{H}}_{{(\mathrm{g})}}}} \right)=218.0\;\mathrm{kJ}\;\mathrm{mo}{{\mathrm{l}}^{{\text{--} 1}}}$ and $\varDelta H_{f,298}^0\left( {\mathrm{C}{{\mathrm{l}}_{{(\mathrm{g})}}}} \right)=121.3\;\mathrm{kJ}\;\mathrm{mo}{{\mathrm{l}}^{{\text{--} 1}}}$ [6, 7].

Table 3 compares the values of $\varDelta H_{f,298}^0$ of the reactants and products of the studied reactions derived on the basis of the total G3-energy calculated for the formation of CH_x_Cl_y_ (a) or for its atomization reaction (b). The results presented show that the calculated values of $\varDelta H_{f,298}^0$ in both approaches (a) and (b) are in very good agreement with the experimental estimates for CH_3_Cl, CH_2_Cl_2_, CH_3_ and CH_2_Cl. However, the heat of reaction ½Cl_2_ → Cl calculated at the G3 level is associated with an error of 2.4 kJ mol^−1^ at 298 K with respect to the experimental estimate. This is the primary cause of the sizable errors in the values of $\varDelta H_{f,298}^0$ calculated by the formation of CH_x_Cl_y_ for molecular structures containing three or more chlorine atoms. The calculations of $\varDelta H_{f,298}^0$ for CH_x_Cl_y_ (y > 2) on the basis of the G3-energy for the atomization reaction reproduce the experimental estimates considerably better [34–37]. The greatest difference between the calculated and experimental value of $\varDelta H_{f,298}^0$ occurs for CCl_4_. However, the values of $\varDelta H_{f,298}^0$ obtained by the latter method are closer to experimental estimates for CCl_4_ as well as the other reactants and products of the studied reactions. This leads to the conclusion that this method is the most suitable approach in calculations of the enthalpy of formation of chlorine-containing species.

**Table 3:** Comparison of experimental $\varDelta \mathrm{H}_{{\mathrm{f}.298}}^0$ (exp.) and theoretical $\varDelta \mathrm{H}_{{\mathrm{f}.298}}^0$ (calc.) values of the enthalpy of formation of the reactants and products of the studied reactions obtained at the G3 level

Molecular system	$\varDelta \mathrm{H}_{{\mathrm{f}.298}}^0$ (calc.) (kJ mol^−1^)		$\varDelta \mathrm{H}_{{\mathrm{f}.298}}^0$ (exp.) (kJ mol^−1^)^c^
	Formation reaction of CH_x_Cl_y_^a^	Atomization reaction^b^	
CH_3_Cl	−80.6	−81.1	−82.0 ± 0.7
CH_2_Cl_2_	−95.9	−93.0	−95.4 ± 2.5
CHCl_3_	−108.7	−102.5	−103.3 ± 1.3
CCl_4_	−112.1	−102.5	−95.8 ± 2.5
CH_3_	145.4	142.6	146.4 ± 0.3
CH_2_Cl	115.9	116.4	117.3 ± 3.1
CHCl_2_	87.1	91.0	89.0 ± 3.0
CCl_3_	64.0	71.2	71.1 ± 2.5
Cl	118.9		121.301 ± 0.008

^a^Calculated from G3-energy for the formation reaction of CH_x_Cl_y_ from the elemental reference compounds

^b^Calculated from G3-energy for the atomization reaction of CH_x_Cl_y_

^c^From Ref. [7] and papers cited therein

**Fig. 2:**
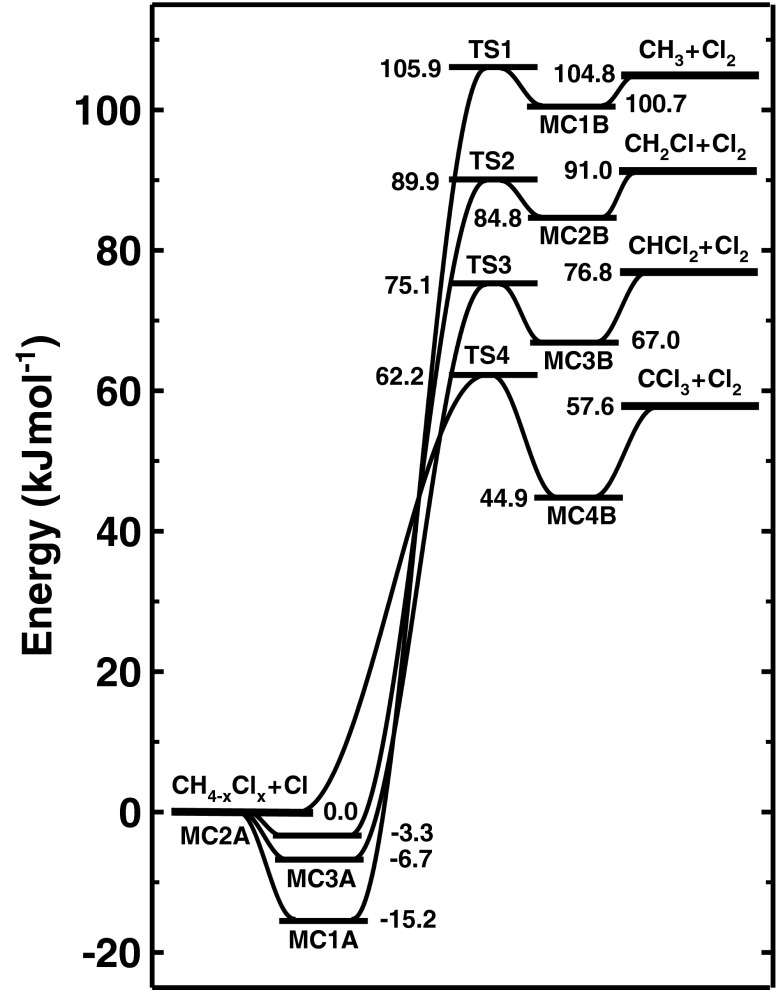
Schematic profiles of the potential energy surfaces for Cl-abstraction reactions from CH_3_Cl, CH_2_Cl_2_, CHCl_3_ and CCl_4_ by atomic chlorine

The reaction enthalpy was calculated directly from the total G3-energy of the reactants and products. The calculated values of reaction enthalpy do not depend on the method used to calculate the enthalpy of formation. Figure 2 shows that all investigated reactions are highly endothermic processes. The reaction enthalpy $\varDelta H_{r,298}^0$ of 107.1 kJ mol^−1^ calculated at the G3 level for reaction CH_3_Cl + Cl ↔ CH_3_ + Cl_2_ at room temperature is in excellent agreement with the experimental value of 107.1 ± 0.9 kJ mol^−1^ [7]. The theoretical value of $\varDelta H_{r,298}^0$ of 92.9 kJ mol^−1^ for CH_2_Cl_2_ + Cl ↔ CH_2_Cl + Cl_2_ is also very close to that of 91.4 ± 5.6 kJ mol^−1^ [7] derived from the experimentally estimated values of $\varDelta H_{f,298}^0$ of the reactants and products. In the case of the reaction CHCl_3_ + Cl ↔ CHCl_2_ + Cl_2_, the calculated value of $\varDelta H_{r,298}^0$ of 77.0 kJ mol^−1^ at 298 K is 6 kJ mol^−1^ higher than the experimental estimate [7]. A more visible difference between the calculated and experimental values of $\varDelta H_{r,298}^0$ occurs for CCl_4_ + Cl ↔ CCl_3_ + Cl_2_. The calculated heat of this reaction of 57.2 kJ mol^−1^ distinctly overestimates the experimental value of 45.6 kJ mol^−1^ [7] derived at room temperature. This is due to the sizable difference between the theoretical and experimental values of the enthalpy of formation of CCl_4_.

### Reaction mechanism

The mechanism of the reactions under investigation appears to be complex and consists of some consecutive elementary processes related with the formation of loosely bound intermediate complexes. The profiles of the potential energy surface are shown in Fig. 2. All reactions studied are highly endothermic. Except for reaction CCl_4_ + Cl, the mechanism of Cl-abstraction proceeds in accordance with a three-step reaction mechanism

4 $$ \mathrm{C}{{\mathrm{H}}_{{4-\mathrm{x}}}}\mathrm{C}{{\mathrm{l}}_{\mathrm{x}}} + \mathrm{C}\mathrm{l}\leftrightarrows \mathrm{C}{{\mathrm{H}}_{{4-\mathrm{x}}}}\mathrm{C}{{\mathrm{l}}_{\mathrm{x}}}\ldots \mathrm{C}\mathrm{l}\leftrightarrows \mathrm{C}{{\mathrm{H}}_{{4-\mathrm{x}}}}\mathrm{C}{{\mathrm{l}}_{{\mathrm{x}-1}}}\ldots \mathrm{C}{{\mathrm{l}}_2}\to \mathrm{C}{{\mathrm{H}}_{{4-\mathrm{x}}}}\mathrm{C}{{\mathrm{l}}_{{\mathrm{x}-1}}}+\mathrm{C}{{\mathrm{l}}_2} $$

where x = 1,2 and 3. The first and third elementary processes are recombination and unimolecular dissociation, while the second is related to an energy barrier. The pre-reaction adducts formed in the first elementary step are loose molecular complexes. The most stable structure is the adduct CH_3_Cl…Cl denoted as MC1A. Its dissociation energy to reactants is 15.2 kJ mol^−1^. The other pre-reaction complexes are considerably less bonded. The next elementary step leads, via TSx, to the molecular complex MCxB, which dissociates to the final channel products, CH_4−x_Cl_x−1_ + Cl_2_. The calculated energy barriers for the second step are high, which implies small values of the rate constants for the studied reactions at ambient temperature. It is interesting that the height of the energy barrier is very close to the reaction heat, and both decrease as the number of chlorine atoms in the reactant molecule increases.

In the case of the reactions CCl_4_ + Cl, the Cl-abstraction process requires only two elementary steps as

5 $$ \mathrm{C}\mathrm{C}{1_4}+\mathrm{C}1\leftrightarrows \mathrm{C}\mathrm{C}{1_3}\ldots \mathrm{C}{1_2}\to \mathrm{C}\mathrm{C}{1_3}+\mathrm{C}{1_2} $$

The intermediate complex, CCl_3_…Cl_2_ denoted as MC4B formed in the first elementary step dissociates into the final reaction products, trichloromethyl radical CCl_3_ and molecular chlorine Cl_2_. The energy barrier for this reaction of 62.2 kJ mol^−1^ is the lowest among the reactions studied. Therefore, the reaction CCl_4_ + Cl should be the fastest process among the reactions under investigation.

### Rate constant calculations

There are some theoretical kinetic models for describing the kinetics of a bimolecular reaction, which involve the formation of intermediate molecular complexes [21, 38, 39]. Assuming that the intermediates formed are loosely bound complexes, their collisional stabilization can, at a first approximation, be omitted in the description of the reaction rate. A method for the rate constant calculation for a bimolecular reaction that proceeds through the formation of pre- (MCxA) and post-reaction (MCxB) complexes has been applied successfully to describe the kinetics of many H-abstraction reactions [21–23]. The general equation, which takes into account the rotational energy, is derived from RRKM theory. According to this formalism, the rate coefficient *k* for the three-step reaction mechanism, such as for Eq. (4) with formation of the pre-reaction and post-reaction adducts, can be expressed as

6 $$ \begin{array}{*{20}c} \hfill k=\frac{z}{{h{Q_{RCl }}{Q_{Cl }}}}\int\limits_{{{V_{TSx}}}}^{\infty } {\sum\limits_J {{W_{MCxA }}\left( {E,J} \right)\times \frac{{{W_{TSx }}\left( {E,J} \right)}}{{{W_{MCxA }}\left( {E,J} \right)+{W_{TSx }}\left( {E,J} \right)}}} } \times \\ \hfill \frac{{{W_{MCxB }}\left( {E,J} \right)}}{{{W_{MCxB }}\left( {E,J} \right)+{W_{TSx }}\left( {E,J} \right)}}\times \exp \left( {-E/RT} \right)dE \\\end{array} $$

where *Q*_RCl_ and *Q*_Cl_ are the partition functions of chloromethane CH_4−x_Cl_x_ (x = 1,2,3 and 4) and atomic chlorine, respectively, with the center of mass partition function factored out of the product *Q*_RCl_ *Q*_Cl_ and included in *z* together with the partition functions of those inactive degrees of freedom that are not considered by the sums of the states under the integral. *V*_TSx_ is the threshold energy towards the reactants CH_4−x_Cl_x_ + Cl, whereas *W*_TSx_(*E*,*J*), *W*_MCxA_(*E*,*J*), and *W*_MCxB_(*E*,*J*) denote the sum of the states at energy less than or equal to *E* and with angular momentum *J* for the transition state TSx and the activated complexes for the unimolecular dissociations of MCxA and MCxB, respectively. All computational effort is then related to calculating the sum of the states, *W*(*E*,*J*) This calculation depends on the level at which the conservation of angular momentum is considered and is discussed in detail in Refs. [21, 22].

Equation 6 can be used directly in the description of kinetics of the reactions CH_3_Cl + Cl, CH_2_Cl_2_ + Cl and CHCl_3_ + Cl. In the case of the two-step mechanism such as for the reaction CCl_4_ + Cl , one must replace *W*_MC4A_(*E*,*J*) by *W*_TS4_(*E*,*J*) and omit the first fraction under the integral in Eq. (6).

The results of direct calculations [13, 20–22] show that the dominant contribution to the rate constant is given by states with energy *E* not higher than *V*_TSx_ + 3RT. In the case of a sizable (compared with RT) energy barrier *V*_TSx_, the value of the product of the microcanonical branching fractions at an energy slightly higher than *V*_TSx_ becomes close to unity and the TST rate constant *k*_TST_ is then a good approximation of the exact rate coefficient, especially at ambient temperatures [13, 20–22].

### Reaction system CH_3_Cl + Cl

The values of the calculated rate constants are given in Table 4. The height of the energy barrier is clearly the major factor determining the magnitude of the rate constant and its dependence on temperature. Figure 2 shows that the Cl-abstraction from CH_3_Cl by Cl atoms is related with a high energy barrier of 106 kJ mol^−1^. The calculated value of the rate constant at 298 K is of 4.5 × 10^−30^ cm^3^molecule^−1^ s^−1^. This value is 17 orders of magnitude lower than the rate constant for the competitive reaction of H-abstraction from CH_3_Cl by atomic chlorine [40]. This is the major reason of the lack of experimental measurements of rate constants for Cl-abstraction from CH_3_Cl. The kinetics of the reverse reaction CH_3_ + Cl_2_ are considerably better recognized [4–6]. The values of the rate constant for this reaction calculated via the equilibrium constant obtained theoretically are also given in Table 4. The values of the rate constants, *k*(CH_3_Cl + Cl) and *k*(CH_3_ + Cl_2_) calculated in the temperature range of 200–3,000 K, can be expressed as:

**Table 4:** Rate constant calculated for the Cl-abstraction reactions CH_4−x_Cl_x_ + Cl → CH_4−x_Cl_x−1_ + Cl_2_ and their reverse processes

T	CH_3_Cl + Cl ↔ CH_3_ + Cl_2_				CH_2_Cl_2_ + Cl ↔ CH_2_Cl + Cl_2_				CHCl_3_ + Cl ↔ CHCl_2_ + Cl_2_				CCl_4_ + Cl ↔ CCl_3_ + Cl_2_			
	*k*(CH_3_Cl + Cl)	*k*_TST_(CH_3_Cl + Cl)	logK_p_	*k*(CH_3_ + Cl_2_)	*k*(CH_2_Cl_2_ + Cl)	*k*_TST_(CH_2_Cl_2_ + Cl)	logK_p_	*k*(CH_2_Cl + Cl_2_)	*k*(CHCl_3_ + Cl)	*k*_TST_(CHCl_3_ + Cl)	logK_p_^a^	*k*(CHCl_2_ + Cl_2_)	*k*(CCl_4_ + Cl)	*k*_TST_(CCl_4_ + Cl)	logK_p_	*k*(CCl_3_ + Cl_2_)
(K)	(cm^3^molecule^−1^ s^−1^)	(cm^3^molecule^−1^ s^−1^)		(cm^3^molecule^−1^ s^−1^)	(cm^3^molecule^−1^ s^−1^)	(cm^3^molecule^−1^ s^−1^)		(cm^3^molecule^−1^ s^−1^)	(cm^3^molecule^−1^ s^−1^)	(cm^3^molecule^−1^ s^−1^)		(cm^3^molecule^−1^ s^−1^)	(cm^3^molecule^−1^ s^−1^)	(cm^3^molecule^−1^ s^−1^)		(cm^3^molecule^−1^ s^−1^)
200	2.21 × 10^−39^	2.44 × 10^−39^	−26.8131	1.39 × 10^−12^	2.60 × 10^−35^	3.24 × 10^−35^	−22.0543	2.83 × 10^−13^	4.41 × 10^−31^	4.41 × 10^−31^	−16.1416	6.10 × 10^−15^	1.67 × 10^−27^	1.68 × 10^−27^	−12.4458	5.75 × 10^−15^
250	9.42 × 10^−34^	1.02 × 10^−33^	−21.2130	1.51 × 10^−12^	1.73 × 10^−30^	2.00 × 10^−30^	−17.2029	2.70 × 10^−13^	4.64 × 10^−27^	4.66 × 10^−27^	−12.3986	1.16 × 10^−14^	3.87 × 10^−24^	3.91 × 10^−24^	−9.4180	1.21 × 10^−14^
298	4.49 × 10^−30^	4.81 × 10^−30^	−17.5717	1.65 × 10^−12^	2.42 × 10^−27^	2.70 × 10^−27^	−14.0551	2.71 × 10^−13^	1.98 × 10^−24^	2.00 × 10^−24^	−9.9802	1.89 × 10^−14^	6.18 × 10^−22^	6.29 × 10^−22^	−7.4703	2.11 × 10^−14^
300	5.90 × 10^−30^	6.31 × 10^−30^	−17.4547	1.66 × 10^−12^	3.06 × 10^−27^	3.41 × 10^−27^	−13.9541	2.71 × 10^−13^	2.41 × 10^−24^	2.43 × 10^−24^	−9.9033	1.92 × 10^−14^	7.28 × 10^−22^	7.41 × 10^−22^	−7.4080	2.16 × 10^−14^
350	3.26 × 10^−27^	3.48 × 10^−27^	−14.7538	1.83 × 10^−12^	6.72 × 10^−25^	7.40 × 10^−25^	−11.6244	2.80 × 10^−13^	2.20 × 10^−22^	2.25 × 10^−22^	−8.1223	2.91 × 10^−14^	3.20 × 10^−20^	3.30 × 10^−20^	−5.9800	3.48 × 10^−14^
400	3.92 × 10^−25^	4.18 × 10^−25^	−12.7171	2.03 × 10^−12^	3.98 × 10^−23^	4.38 × 10^−23^	−9.8716	2.95 × 10^−13^	6.73 × 10^−21^	6.97 × 10^−21^	−6.7878	4.13 × 10^−14^	5.65 × 10^−19^	5.92 × 10^−19^	−4.9152	5.20 × 10^−14^
450	1.69 × 10^−23^	1.81 × 10^−23^	−11.1257	2.24 × 10^−12^	9.79 × 10^−22^	1.09 × 10^−21^	−8.5049	3.12 × 10^−13^	9.87 × 10^−20^	1.04 × 10^−19^	−5.7513	5.57 × 10^−14^	5.37 × 10^−18^	5.74 × 10^−18^	−4.0921	7.34 × 10^−14^
500	3.53 × 10^−22^	3.81 × 10^−22^	−9.8479	2.48 × 10^−12^	1.29 × 10^−20^	1.46 × 10^−20^	−7.4096	3.32 × 10^−13^	8.61 × 10^−19^	9.25 × 10^−19^	−4.9239	7.23 × 10^−14^	3.30 × 10^−17^	3.61 × 10^−17^	−3.4379	9.91 × 10^−14^
600	3.56 × 10^−20^	3.94 × 10^−20^	−7.9238	2.98 × 10^−12^	6.48 × 10^−19^	7.57 × 10^−19^	−5.7646	3.77 × 10^−13^	2.30 × 10^−17^	2.58 × 10^−17^	−3.6873	1.12 × 10^−13^	5.16 × 10^−16^	5.95 × 10^−16^	−2.4664	1.63 × 10^−13^
700	1.01 × 10^−18^	1.15 × 10^−18^	−6.5450	3.53 × 10^−12^	1.10 × 10^−17^	1.34 × 10^−17^	−4.5896	4.28 × 10^−13^	2.46 × 10^−16^	2.91 × 10^−16^	−2.8095	1.59 × 10^−13^	3.76 × 10^−15^	4.59 × 10^−15^	−1.7824	2.44 × 10^−13^
800	1.28 × 10^−17^	1.51 × 10^−17^	−5.5099	4.13 × 10^−12^	9.39 × 10^−17^	1.20 × 10^−16^	−3.7099	4.82 × 10^−13^	1.49 × 10^−15^	1.85 × 10^−15^	−2.1559	2.13 × 10^−13^	1.68 × 10^−14^	2.19 × 10^−14^	−1.2768	3.39 × 10^−13^
900	9.40 × 10^−17^	1.15 × 10^−16^	−4.7054	4.77 × 10^−12^	5.06 × 10^−16^	6.80 × 10^−16^	−3.0280	5.41 × 10^−13^	6.10 × 10^−15^	8.02 × 10^−15^	−1.6519	2.74 × 10^−13^	5.45 × 10^−14^	7.58 × 10^−14^	−0.8895	4.47 × 10^−13^
1000	4.70 × 10^−16^	5.96 × 10^−16^	−4.0630	5.44 × 10^−12^	1.97 × 10^−15^	2.78 × 10^−15^	−2.4848	6.03 × 10^−13^	1.90 × 10^−14^	2.64 × 10^−14^	−1.2523	3.39 × 10^−13^	1.40 × 10^−13^	2.08 × 10^−13^	−0.5844	5.66 × 10^−13^
1500	6.44 × 10^−14^	9.90 × 10^−14^	−2.1559	9.26 × 10^−12^	1.24 × 10^−13^	2.21 × 10^−13^	−0.8807	9.50 × 10^−13^	6.02 × 10^−13^	1.09 × 10^−12^	−0.0859	7.34 × 10^−13^	2.39 × 10^−12^	4.96 × 10^−12^	0.2925	1.27 × 10^−12^
2000	8.01 × 10^−13^	1.48 × 10^−12^	−1.2290	1.37 × 10^−11^	1.04 × 10^−12^	2.27 × 10^−12^	−0.1083	1.35 × 10^−12^	3.49 × 10^−12^	8.02 × 10^−12^	0.4641	1.20 × 10^−12^	9.71 × 10^−12^	2.77 × 10^−11^	0.6943	2.02 × 10^−12^
2500	3.73 × 10^−12^	8.10 × 10^−12^	−0.6918	1.85 × 10^−11^	3.85 × 10^−12^	9.96 × 10^−12^	0.3353	1.79 × 10^−12^	1.01 × 10^−11^	2.87 × 10^−11^	0.7732	1.71 × 10^−12^	2.19 × 10^−11^	8.38 × 10^−11^	0.9135	2.74 × 10^−12^
3000	1.05 × 10^−11^	2.65 × 10^−11^	−0.3470	2.35 × 10^−11^	9.31 × 10^−12^	2.81 × 10^−11^	0.6173	2.26 × 10^−12^	2.06 × 10^−11^	7.08 × 10^−11^	0.9652	2.23 × 10^−12^	3.69 × 10^−11^	1.85 × 10^−10^	1.0450	3.40 × 10^−12^

^a^The equilibrium constant K_p_ calculated for the reaction enthalpy corrected by −6.0 kJ mol^−1^ to obtain value of $\varDelta \mathrm{H}_{{\mathrm{f}.298}}^0$ of 71.0 kJ mol^−1^ (see text)

7 $$ \begin{array}{*{20}c} {k\left( {\mathrm{C}{{\mathrm{H}}_3}\mathrm{Cl}+\mathrm{Cl}} \right)=2.08\times {10^{-11 }}\times {{{\left( {{{\mathrm{T}} \left/ {300 } \right.}} \right)}}^{1.63 }}\times\exp \left( {{-12780 \left/ {\mathrm{T}} \right.}} \right)}{\mathrm{c}{{\mathrm{m}}^3}\mathrm{molecul}{{\mathrm{e}}^{-1 }}{{\mathrm{s}}^{-1 }}} \\ \end{array} $$

8 $$ \begin{array}{*{20}c} {k\left( {\mathrm{C}{{\mathrm{H}}_3}+\mathrm{C}{{\mathrm{l}}_2}} \right)=6.70\times {10^{-13 }}\times {{{\left( {{T \left/ {300 } \right.}} \right)}}^{1.51 }}\times \exp \left( {{270 \left/ {\mathrm{T}} \right.}} \right)}{\mathrm{c}{{\mathrm{m}}^3}\mathrm{molecul}{{\mathrm{e}}^{-1 }}{{\mathrm{s}}^{-1 }}} \\ \end{array} $$

**Fig. 3:**
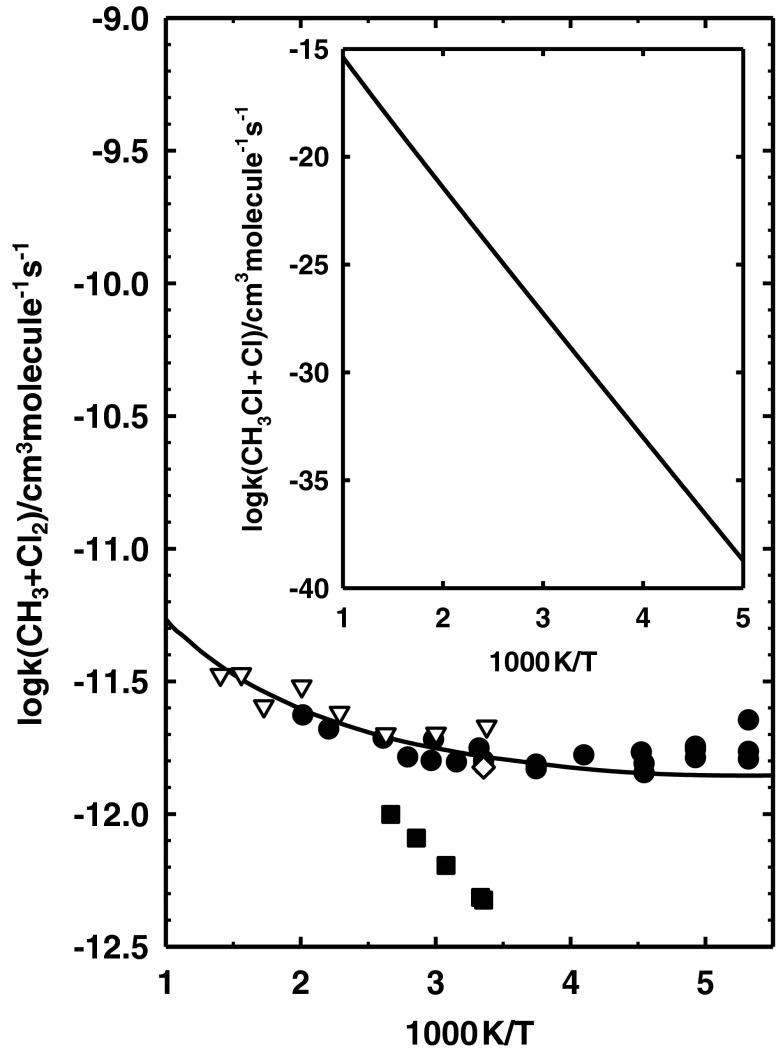
Arrhenius plot for the reaction CH_3_ + Cl_2_ → CH_3_Cl + Cl comparing the available results of kinetic measurements of Eskola et al. [41] (*black circles*), Timonen et al. [42] (*black squares*), Timonen and Gutman [43] (*white triangles*), and Kovalenko and Leone [44] (*white diamonds*) with those obtained theoretically in this study. *Solid line* Plot of Eq. (8). *Inset* Temperature dependence of *k*(CH_3_Cl + Cl) from Eq. (7)

The calculated values of *k*(CH_3_ + Cl_2_) are compared with available experimental results in Fig. 3. The inset shows the temperature dependence of the calculated *k*(CH_3_Cl + Cl). The room temperature value of the rate constant *k*(CH_3_Cl + Cl) is very low: 2.0 × 10^−24^ cm^3^molecule^−1^ s^−1^. However, the values of *k*(CH_3_Cl + Cl) depend strongly on temperature. The increase in temperature from 200 K to 1,000 K results in a rise in the value of *k*(CH_3_Cl + Cl) by over 20 orders of magnitude. The calculated values of *k*(CH_3_Cl + Cl) are 4.7 × 10^−16^ and 1.1 × 10^−11^ cm^3^molecule^−1^ s^−1^ at 1,000 K and 3,000 K, respectively.

The reverse reaction has been studied experimentally over a wide temperature range. Figure 3 compares our calculated values of *k*(CH_3_ + Cl_2_) with the experimental results of Eskola et al. [41] performed in the range of 188–500 K, Timonen et al. [42] at 298–423 K, Timonen and Gutman [43] at 296–712 K, and those of Kovalenko and Leone [44] studied at 298 K. The results of Timonen et al. [42] are distinctly underestimated. The other experimental measurements are consistent and show only little dispersion. The calculated values of *k*(CH_3_ + Cl_2_) can be considered the best compromise between the available estimates. The predicted temperature dependence of the rate constants, *k*(CH_3_Cl + Cl) and *k*(CH_3_ + Cl_2_) expressed by Eqs. (7) and (8) allows a description of the kinetics of the reactions CH_3_Cl + Cl and CH_3_ + Cl_2_ over a wide temperature range.

### Reaction system CH_2_Cl_2_ + Cl

**Fig. 4:**
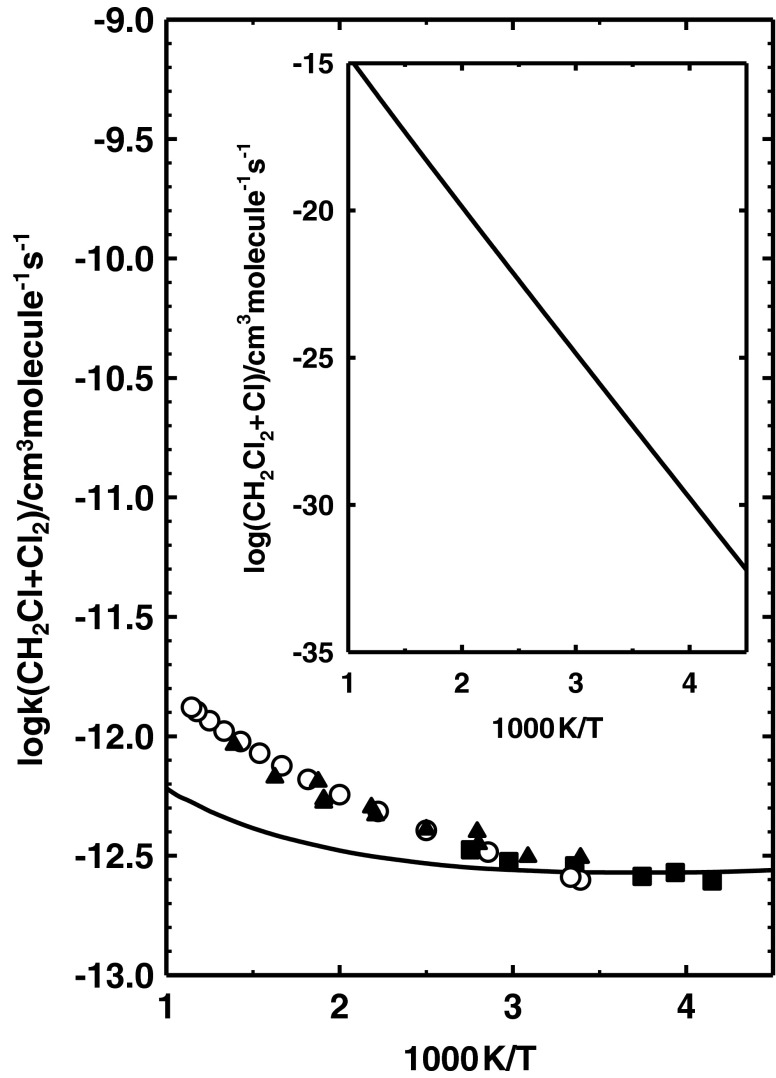
Arrhenius plot for the reaction CH_2_Cl + Cl_2_ → CH_2_Cl_2_ + Cl comparing the available results of kinetic measurements of Seetula [45] (*white circles*), Seetula et al. [46] (*black triangles*) and Eskola et al. [47] by (*black squares*) with those obtained theoretically in this study. *Solid line* Plot of Eq. (10). *Inset* Temperature dependence of *k*(CH_2_Cl_2_ + Cl) from Eq. (9)

The profiles of the potential energy surface presented in Fig. 2 show that Cl abstraction from CH_2_Cl_2_ by Cl atoms is also related to a high energy barrier of 90 kJ mol^−1^. This implies a low values of the rate constants *k*(CH_2_Cl_2_ + Cl). The calculated value of *k*(CH_2_Cl_2_ + Cl) at 298 K of 2.4 × 10^−27^ cm^3^molecule^−1^ s^−1^ is over 1,000 times higher than the value of *k*(CH_3_Cl + Cl) at the same temperature. The height of the energy barrier is lower by 16 kJ mol^−1^ than that calculated for CH_3_Cl + Cl. As a consequence, values of *k*(CH_2_Cl_2_ + Cl) increase a little more weakly with temperature than values of *k*(CH_3_Cl + Cl). The calculated values of *k*(CH_2_Cl_2_ + Cl) are 2.0 × 10^−15^ and 9.3 × 10^−12^ cm^3^molecule^−1^ s^−1^ at 1,000 K and 3,000 K, respectively. To the best of our knowledge there is no experimental information on the kinetics of CH_2_Cl_2_ + Cl. However, the reverse reaction CH_2_Cl + Cl_2_ has been studied experimentally [45–47] in the temperature range 201–873 K. Figure 4 compares our theoretical results with the results of the experimental investigations of Seetula [45], Seetula et al. [46] and Eskola et al. [47] obtained for CH_2_Cl + Cl_2_. The values of the rate constants, *k*(CH_2_Cl_2_ + Cl) and *k*(CH_2_Cl + Cl_2_) calculated in the temperature range of 200–3,000 K, can be expressed as

9 $$ \begin{array}{*{20}c} {k\left( {\mathrm{C}{{\mathrm{H}}_2}\mathrm{C}{{\mathrm{l}}_2}+\mathrm{Cl}} \right)=2.36\times {10^{-11 }}\times {{{\left( {{{\mathrm{T}} \left/ {300 } \right.}} \right)}}^{1.23 }}\times\exp \left( {{-10960 \left/ {\mathrm{T}} \right.}} \right)}{\mathrm{c}{{\mathrm{m}}^3}\mathrm{molecul}{{\mathrm{e}}^{-1 }}{{\mathrm{s}}^{-1 }}} \\ \end{array} $$

10 $$ \begin{array}{*{20}c} {k\left( {\mathrm{C}{{\mathrm{H}}_2}\mathrm{Cl}+\mathrm{C}{{\mathrm{l}}_2}} \right)=7.34\times {10^{-14 }}\times {{{\left( {{{\mathrm{T}} \left/ {300 } \right.}} \right)}}^{1.43 }}\times \exp \left( {{390 \left/ {\mathrm{T}} \right.}} \right)}{\mathrm{c}{{\mathrm{m}}^3}\mathrm{molecul}{{\mathrm{e}}^{-1 }}{{\mathrm{s}}^{-1 }}} \\ \end{array} $$

The results of our calculations reproduce the experimentally estimated values of *k*(CH_2_Cl + Cl_2_) very well at low temperatures, i.e., below 400 K. At higher temperatures, our values of *k*(CH_2_Cl + Cl_2_) are slightly lower compared with the experimental results. The difference between the experimental and theoretical results increases with increasing temperature. The experimental value of (9.22 ± 0.54) × 10^−13^ cm^3^molecule^−1^ s^−1^ obtained by Seetula et al. [46] at 719 K is two times higher than the 4.4 × 10^−13^ cm^3^molecule^−1^ s^−1^ derived from Eq. (10). This may be an effect of the treatment of the lowest degrees of freedom of the transition state TS2 as harmonic vibrations. However, it should be emphasized that the theoretically derived values of *k*(CH_2_Cl + Cl_2_) and *k*(CH_2_Cl_2_ + Cl) describe very well the kinetics of the chlorine abstraction reactions CH_2_Cl_2_ + Cl and CH_2_Cl + Cl_2_ at temperatures not higher than ambient.

### Reaction system CHCl_3_ + Cl

The calculated energy barrier of 75 kJ mol^−1^ for reaction CHCl_3_ + Cl is lower by 15 and 31 kJ mol^−1^ than those derived for CH_2_Cl_2_ + Cl and CH_3_Cl + Cl, respectively. This indicates that chlorine abstraction proceeds more easily from a reactant with a greater number of chlorine atoms. The height of the energy barrier for chlorine abstraction decreases by 15 kJ mol^−1^ for any subsequent chlorine atom inserted into the chloromethane molecule. However, the energy barrier for CHCl_3_ + Cl is still high, which implies a low value of the rate constant *k*(CH_2_Cl_2_ + Cl) at room temperature. The calculated value of *k*(CH_2_Cl_2_ + Cl) at 298 K is of 2.0 × 10^−24^ cm^3^molecule^−1^ s^−1^. This value is over 3 and 6 orders of magnitude higher than the calculated at the same temperature values of *k*(CH_3_Cl + Cl) and *k*(CH_2_Cl_2_ + Cl), respectively. The derived temperature dependence of *k*(CHCl_3_ + Cl) can be expressed in the temperature range 200–3,000 K as,

11 $$ \begin{array}{*{20}c} {k\left( {\mathrm{CHC}{{\mathrm{l}}_3}+\mathrm{Cl}} \right)=5.28\times {10^{-11 }}\times {{{\left( {{{\mathrm{T}} \left/ {300 } \right.}} \right)}}^{0.97 }}\times \exp \left( {{-9200 \left/ {\mathrm{T}} \right.}} \right)}{\mathrm{c}{{\mathrm{m}}^3}\mathrm{molecul}{{\mathrm{e}}^{-1 }}{{\mathrm{s}}^{-1 }}} \\ \end{array} $$

**Fig. 5:**
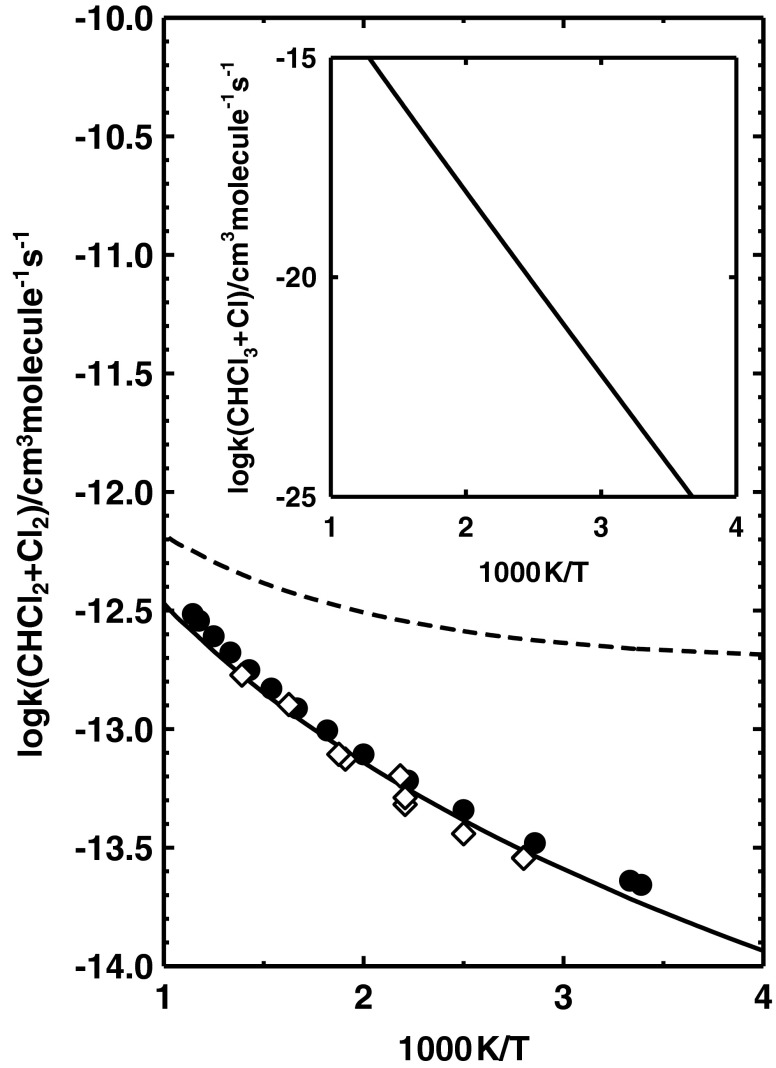
Arrhenius plot for the reaction CHCl_2_ + Cl_2_ → CHCl_3_ + Cl comparing the available results of kinetic measurements of Seetula [45] (*black circles*) and Seetula et al. [46] (*white diamonds*) with those obtained theoretically in this study. The *dashed* and *solid curves *show the temperature dependence of *k*(CHCl_2_ + Cl_2_) derived from the values of the rate constant *k*(CHCl_3_ + Cl) and the equilibrium constant corresponding to the reaction enthalpy at room temperature of 77.0 and 71.0 kJ mol^−1^ (see text), respectively. *Inset* Temperature dependence of *k*(CHCl_3_ + Cl) from Eq. (11)

According to the sizable energy barrier, the reaction CHCl_3_ + Cl becomes important only at very high temperatures. Experimental information is available only for the reverse process, CHCl_2_ + Cl_2_ [45, 46]. Figure 5 shows a comparison of the temperature dependence of the rate constant *k*(CHCl_2_ + Cl_2_) derived theoretically with that from experimental findings [45, 46]. The dashed line denotes the values of the rate constant *k*(CHCl_2_ + Cl_2_) obtained *via* the calculated equilibrium constant. The calculated values of *k*(CHCl_2_ + Cl_2_) distinctly overestimate the experimental results of Seetula [45] and Seetula et al. [46], especially at low temperatures.

It is worth noting that a comparison of the theoretical and experimental values of *k*(CHCl_2_ + Cl_2_) is indeed the only way to verify, albeit indirectly, the calculated values of both *k*(CHCl_2_ + Cl_2_) and *k*(CHCl_3_ + Cl), on the condition that the calculated equilibrium constants are realistic. The calculated reaction enthalpy at room temperature for CHCl_3_ + Cl → CHCl_2_ + Cl_2_ is of 77.0 kJ mol^−1^, whereas the experimentally estimated one is 71.0 kJ mol^−1^ [7]. The correction of the reaction enthalpy by −6.0 kJ mol^−1^ at any temperature should lead to more realistic values of the equilibrium constant, and finally to a more reliable rate constant *k*(CHCl_2_ + Cl_2_). The values of *k*(CHCl_2_ + Cl_2_) after reduction by a factor of exp(6.0 kJ mol^−1^/RT), shown in Fig. 5 (solid line), can be expressed by

12 $$ \begin{array}{*{20}c} {k\left( {\mathrm{CHC}{{\mathrm{l}}_2}+\mathrm{C}{{\mathrm{l}}_2}} \right)=6.81\times {10^{-14 }}\times {{{\left( {{{\mathrm{T}} \left/ {300 } \right.}} \right)}}^{1.60 }}\times \exp \left( {{-370 \left/ {\mathrm{T}} \right.}} \right)}{\mathrm{c}{{\mathrm{m}}^3}\mathrm{molecul}{{\mathrm{e}}^{-1 }}{{\mathrm{s}}^{-1 }}} \\ \end{array} $$

The corrected values of the rate constant, *k*(CHCl_2_ + Cl_2_) reproduce the experimental results very well. Agreement between experimental and calculated values of *k*(CHCl_2_ + Cl_2_) is excellent over a wide temperature range. This also confirms the reliability of the values of the rate constant, *k*(CHCl_3_ + Cl) calculated in this study.

### Reaction system CCl_4_ + Cl

Figure 2 shows that the lowest energy barrier of 62 kJ mol^−1^ was found for the reaction of CCl_4_ + Cl. The calculated value of *k*(CCl_4_ + Cl) of 6.2 × 10^−22^ cm^3^molecule^−1^ s^−1^ at 298 K is over 300 times higher than the value of *k*(CHCl_3_ + Cl) at the same temperature (see Table 4). The sizable energy barrier for CCl_4_ + Cl results in the distinct temperature dependence of *k*(CCl_4_ + Cl). The values of *k*(CCl_4_ + Cl) and *k*(CCl_3_ + Cl_2_) for the reverse reaction calculated via the equilibrium constant can be expressed in the form

13 $$ \begin{array}{*{20}c} {k\left( {\mathrm{CC}{{\mathrm{l}}_4}+\mathrm{Cl}} \right)=1.51\times {10^{-10 }}\times {{{\left( {{{\mathrm{T}} \left/ {300 } \right.}} \right)}}^{0.58 }}\times \exp \left( {{-7790 \left/ {\mathrm{T}} \right.}} \right)}{\mathrm{c}{{\mathrm{m}}^3}\mathrm{molecul}{{\mathrm{e}}^{-1 }}{{\mathrm{s}}^{-1 }}} \\ \end{array} $$

14 $$ \begin{array}{*{20}c} {k\left( {\mathrm{CC}{{\mathrm{l}}_3}+\mathrm{C}{{\mathrm{l}}_2}} \right)=1.43\times {10^{-13 }}\times {{{\left( {{{\mathrm{T}} \left/ {300 } \right.}} \right)}}^{1.52 }}\times \exp \left( {{-550 \left/ {\mathrm{T}} \right.}} \right)}{\mathrm{c}{{\mathrm{m}}^3}\mathrm{molecul}{{\mathrm{e}}^{-1 }}{{\mathrm{s}}^{-1 }}} \\ \end{array} $$

The kinetics of reaction CCl_4_ + Cl have been studied experimentally by Seetula [45] and DeMare and Huybrechts [48]. Their investigations were, however, performed in different temperature ranges, and the reported temperature dependencies of *k*(CCl_4_ + Cl) expressed in the Arrhenius’ form show distinct differences in both the pre-exponential factor and the activation energy, which makes it difficult to compare the experimental and theoretical values of *k*(CCl_4_ + Cl). There is no experimental information on the kinetics of the reverse reaction CCl_3_ + Cl_2_. The reaction CCl_4_ + Cl is one of the processes involved in the pyrolysis of CCl_4_ in the gas-phase. Huybrechts et al. [49] studied the pyrolysis of CCl_4_ in terms of modeling by computer simulations and optimizations of the kinetic parameters of the elementary processes taking part in the mechanism of the investigated pyrolysis. The Arrhenius parameters derived from the kinetic model of Huybrechts et al. [49] allow a description of the kinetics of the reaction CCl_4_ + Cl in the temperature range 300–800 K. The value of 4.9 × 10^−22^ cm^3^molecule^−1^ s^−1^ derived at 300 K by Huybrechts et al. [49] is lower than our estimate of 7.3 × 10^−22^ cm^3^molecule^−1^ s^−1^. The similar difference between our result for the value of *k*(CCl_4_ + Cl) and that derived by Huybrechts et al. [49] is maintained at temperatures below 800 K. This agreement can be considered as satisfactory taking into account the uncertainties of the kinetic modeling procedure.

## Summary

The main aim of the present study was to perform a theoretical analysis of the kinetics of chlorine abstraction from chlorinated methanes, CH_3_Cl, CH_2_Cl_2_, CHCl_3_ and CCl_4_ by chlorine atoms. Theoretical investigations based on ab initio calculations of the CH_4−x_Cl_x_ + Cl → CH_4−x_Cl_x−1_ + Cl_2_ (x = 1,2,3 and 4) reaction systems at the G3 level were performed to gain insight into the reaction mechanism. Kinetic information on these reactions is very limited. The results of the calculations also allow an estimation of the reaction energetics and the molecular properties of the structures taking part in the reaction mechanism.

The calculated values of the enthalpy of formation of the reactants and products are in good agreement with the reported values estimated experimentally. All the studied reactions are strongly endothermic processes, with calculated values of reaction enthalpy at 298 K of 107.1, 92.9, 77.0 and 57.2 kJmol^−1^ for CH_3_Cl + Cl, CH_2_Cl_2_ + Cl, CHCl_3_ + Cl and CCl_4_ + Cl, respectively. The calculated profiles of the potential energy surface show that the mechanism of the reactions studied is complex and that Cl-abstraction proceeds via the formation of intermediate complexes. The multi-step reaction mechanism consists of two elementary steps in the case of CCl_4_ + Cl, and three steps for the other reactions. The heights of the energy barrier relative to the Cl-abstraction by Cl atoms from CH_3_Cl, CH_2_Cl_2_, CHCl_3_ and CCl_4_ are 106, 90, 75 and 62 kJmol^−1^, respectively. The differences in energy barriers are reflected in the values of the rate constants. The rate constants calculated at 298 K are 4.5 × 10^−30^, 2.4 × 10^−27^, 2.0 × 10^−24^ and 6.2 × 10^−22^ cm^3^molecule^−1^ s^−1^ for reactions CH_3_Cl/CH_2_Cl_2_/CHCl_3_/CCl_4_ + Cl, respectively. The rate constants for the reverse reactions CH_3_/CH_2_Cl/CHCl_2_/CCl_3_ + Cl_2_ have also been calculated using the equilibrium constants derived theoretically. The ordering of the values of the calculated rate constants for the reverse reactions is quite the opposite of their counterparts in the forward direction. The highest value of 1.7 × 10^−12^ cm^3^molecule^−1^ s^−1^ at 298 K is found for *k*(CH_3_ + Cl_2_), the lowest one is *k*(CCl_3_ + Cl_2_) = 2.1 × 10^−14^ cm^3^molecule^−1^ s^−1^. The calculated values of the rate constants describe the kinetics of the reverse reactions well. An especially good agreement between the calculated and reported values of the rate constants was reached for reactions CH_3_ + Cl_2_, CH_2_Cl + Cl_2_ and CHCl_2_ + Cl_2_. In the temperature range of 200–400 K, the theoretically derived kinetic parameters for these reactions allow the reaction kinetics to be described with an accuracy no worse than that given by various kinetic data evaluations.

This confirms the reliability of the theoretically derived kinetic expressions, which represent a substantial supplement to the kinetic data necessary for the description and modeling of the complex gas-phase reactions of importance in combustion and atmospheric chemistry.
